# Quantification and Selection of Ictogenic Zones in Epilepsy Surgery

**DOI:** 10.3389/fneur.2019.01045

**Published:** 2019-10-01

**Authors:** Petroula Laiou, Eleftherios Avramidis, Marinho A. Lopes, Eugenio Abela, Michael Müller, Ozgur E. Akman, Mark P. Richardson, Christian Rummel, Kaspar Schindler, Marc Goodfellow

**Affiliations:** ^1^Living Systems Institute, University of Exeter, Exeter, United Kingdom; ^2^EPSRC Centre for Predictive Modelling in Healthcare, University of Exeter, Exeter, United Kingdom; ^3^College of Engineering, Mathematics and Physical Sciences, University of Exeter, Exeter, United Kingdom; ^4^Research Computing Services, University of Cambridge, Cambridge, United Kingdom; ^5^Wellcome Trust Centre for Biomedical Modelling and Analysis, University of Exeter, Exeter, United Kingdom; ^6^Institute of Psychiatry, Psychology and Neuroscience, King's College London, London, United Kingdom; ^7^Support Center for Advanced Neuroimaging, University Institute for Diagnostic and Interventional Neuroradiology, Inselspital, Bern University Hospital, University of Bern, Bern, Switzerland; ^8^Department of Neurology, Inselspital, University Hospital Bern, Bern, Switzerland

**Keywords:** epilepsy surgery, brain networks, ictogenesis, graph theory, optimization, genetic algorithm

## Abstract

Network models of brain dynamics provide valuable insight into the healthy functioning of the brain and how this breaks down in disease. A pertinent example is the use of network models to understand seizure generation (ictogenesis) in epilepsy. Recently, computational models have emerged to aid our understanding of seizures and to predict the outcome of surgical perturbations to brain networks. Such approaches provide the opportunity to quantify the effect of removing regions of tissue from brain networks and thereby search for the optimal resection strategy. Here, we use computational models to elucidate how sets of nodes contribute to the ictogenicity of networks. In small networks we fully elucidate the ictogenicity of all possible sets of nodes and demonstrate that the distribution of ictogenicity across sets depends on network topology. However, the full elucidation is a combinatorial problem that becomes intractable for large networks. Therefore, we combine computational models with a genetic algorithm to search for minimal sets of nodes that contribute significantly to ictogenesis. We demonstrate the potential applicability of these methods in practice by identifying optimal sets of nodes to resect in networks derived from 20 individuals who underwent resective surgery for epilepsy. We show that they have the potential to aid epilepsy surgery by suggesting alternative resection sites as well as facilitating the avoidance of brain regions that should not be resected.

## Introduction

Approximately 30% of people with epilepsy have refractory seizures, i.e. their seizures cannot be controlled by medication (1, 2). In these cases, the surgical removal or disconnection of the putative “epileptogenic zone” (EZ), i.e. the region of brain tissue thought to be indispensable for the generation of seizures, is a potential therapeutic option that can alleviate seizures in many cases (3, 4). The epileptogenic zone is currently defined retrospectively: diverse information is integrated by clinical teams to define targets for resection, and if seizure freedom is achieved after surgery, the EZ is assumed to have been removed (3). Unfortunately, post-operative seizure freedom rates are currently sub-optimal and not everyone who could potentially benefit from surgery is identified as a candidate (5–9). In order to improve the success of epilepsy surgery and widen its potential usage, a better understanding of the mechanisms of seizure generation is required, and improved, quantitative methods to prospectively map the EZ need to be developed (10, 11).

Computational studies of seizure generation in large-scale brain networks with the aim to inform epilepsy surgery have recently begun to emerge (11–14). In an early such study we introduced a quantitative framework for prospectively evaluating the effect that surgical removal of tissue would have (11). The framework proceeds by first mapping an ictogenic network i.e. a set of brain regions, together with connections between them, that are important for the generation of seizures. A dynamic model is then applied to this network in order to simulate epileptiform dynamics and thereby quantify the seizure generating capability of the network. This is captured in a quantity called Brain Network Ictogenicity (*BNI*) that can be measured from simulations of the model (15). Crucially, measuring the *BNI* for a network provides a baseline against which the effect of perturbations, such as the removal of nodes (which is a proxy for surgery), can be quantified. We recently showed that our model could accurately delineate surgeries that resulted in seizure freedom from those that did not (11). Subsequent studies have added evidence for the potential use of models to guide epilepsy surgery (12, 13). Using models to quantify ictogenicity opens up avenues for the improvement of pre-surgical mapping. For example, putative resections can be quantitatively compared *in silico*, and resections providing large reductions in ictogenicity (i.e. substantial reduction in seizure occurrence) can be sought. This in itself leads to a re-imagining of the concept of the epileptogenic zone for pre-surgical planning: rather than searching for correlates of a region of tissue that should render a person seizure free, we can quantify the seizure reducing capabilities of removing many alternative sets of nodes in a network prospectively, *in silico*.

Here, we use a quantity called Set Ictogenicity (*SI*) [previously introduced as Δ*BNI* (11)] to represent the extent to which ictogenicity is reduced when a given set of nodes is removed from a network. *SI* can be quantified for any potential set of nodes in a resection using the framework described above. Once the effect of removing a set of nodes can be quantified, one can compare the effect of removing different sets, and choose the set with the minimal number of nodes that would yield the largest reduction in ictogenicity (i.e. find the set with largest *SI*, which would be designated as a putative EZ). However, to ensure an optimal solution is uncovered, one would have to evaluate the *SI* of all possible combinations of sets of nodes within a given network. Unfortunately, this is a combinatorial optimization problem in which the number of sets to search through quickly becomes intractable, even for moderately sized networks (16, 17). Though in practice constraints on the location of resections may exist, alternative strategies to exhaustive searches have to be explored. One could use heuristics that are quick but do not guarantee finding an optimum (18, 19). For example, previous approaches (11, 12, 20) applied heuristics to attempt to identify a set of nodes that optimally reduces the network ictogenicity. However, the selection of nodes of that set was solely based on the contribution of single nodes to seizure generation. In other application areas, combinatorial problems have previously been approached using global non-deterministic search strategies, like simulated annealing (21), evolution strategies (22), genetic algorithms (16, 17) and particle swarm optimization (23). The deployment of such approaches to brain networks would enable us to gain a deeper insight into the way that ictogenesis is distributed throughout networks and facilitate the development of optimal strategies for epilepsy surgery.

Here we use computational models to study the ictogenicity of sets of nodes within a network. We use artificial networks to explore how *SI* varies across sets of nodes within networks of different topologies and to characterize the relationship between common graph metrics and *SI*. To facilitate the search for optimal resections, we develop and validate a multi-objective genetic algorithm to uncover sets of nodes that optimally reduce the ictogenicity of a network. In addition, we apply the methodology to a cohort of 20 people who underwent epilepsy surgery. Finally, we discuss the potential benefits of these approaches to both enhance our understanding of epilepsy and advance pre-surgical planning in practice.

## Materials and Methods

### Simulation of Brain Dynamics

**Figure 1:**
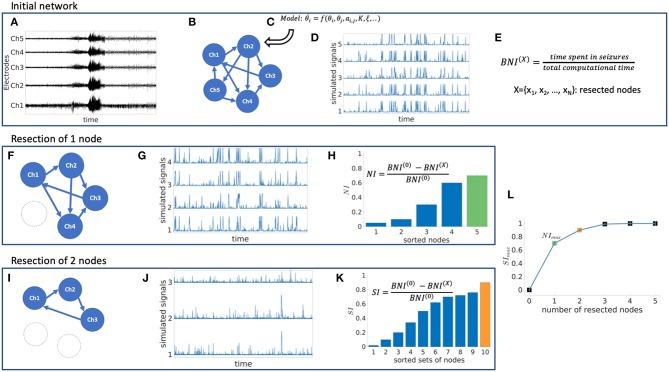
Schematic representation of the mathematical framework. From intracranial recordings **(A)** we construct a functional network **(B)**. We then place a mathematical model on each node **(C)**, simulate signals from the model **(D)** and calculate brain network ictogenicity **(E)**. Then perturbations are applied to the network by removing individual nodes **(F)** or set of nodes **(I)**. Using the simulated signals from the perturbed networks **(G,J)**, Node Ictogenicity (*NI*) and Set Ictogenicity (*SI*) are calculated for all possible combinations of resected nodes [panels **(H,K)**, respectively]. Finally, for every number of resected nodes the set that contributes most to the seizure generation is the one with the maximum *SI* **(H,K,L)**. **(L)** Illustrates the *SI* of the most ictogenic sets split out by resection size.

We use a mathematical modeling framework to simulate and predict the outcome of epilepsy surgery (11). The framework uses intracranial electroencephalographic (iEEG) recordings (Figure 1A) to construct functional networks (Figure 1B), where nodes are associated with electrodes and edges denote interrelations between the recorded signals. We use a surrogate corrected version of mutual information (10, 24) that detects non-linear dependencies in excess of linear relationships to define weighted edges of the network (see Supplementary Text S1 and Supplementary Figure 1). We then place a mathematical model on each node (Figure 1C). We use the canonical theta model (20, 25) which has previously been shown to give a good approximation of the response of the Wendling neural mass model (26) to node removal perturbations (20). A key assumption underlying this approximation is that the neural mass model is operating in the vicinity of a saddle node on invariant circle (SNIC) bifurcation, which has previously been used to model epileptiform dynamics (11, 26, 27). Nodes can display transitions between “normal activity” (stable fixed point) and “epileptiform dynamics” (stable limit cycle) through a saddle node on invariant circle (SNIC) bifurcation. The dynamics of node *j* (*j* = 1, …, *N*, where N is the number of nodes in the network) is represented by its phase θ_*j*_ and obeys

(1) $$\begin{array}{l} {\dot{\theta }}_{j}\left(t\right)=\left(1-\cos\theta_{j}\left(t\right)\right)+\left(1+\cos\theta_{j}\left(t\right)\right)I_{j}\left(t\right) \end{array}$$

where *I*_*j*_(*t*) is the input current that the node receives from the other nodes in the network,

(2) $$\begin{array}{l} I_{j}\left(t\right)=I_{o}+\xi_{j}\left(t\right)+\;\frac{K}{N}\;\sum_{i=1}^{N}a_{ij}\left[1-\cos\left(\theta_{i}\left(t\right)-\theta_{i}^{s}\left(t\right)\right)\right]. \end{array}$$

In Equation (2) the term *I*_*o*_ + ξ_*j*_ is white uncorrelated Gaussian noise with mean *I*_*o*_ = −1.2 and standard deviation 0.6, as used in previous studies (20). *a*_*ij*_ is the (*i, j*) th entry of the adjacency matrix (i.e., the weighted matrix that represents the functional network), *K* is a global scaling factor that scales the network interactions compared to the noise, and $\theta_{i}^{s}$ is the stable fixed point of node *i* ($\dot{\theta_{i}}=0$ at ξ_*i*_ = *K* = 0). For the integration of Equation (1) we use the Euler-Maruyama method with step size 0.01.

### Quantification of Ictogenicity

The theta model enables us to simulate brain dynamics on a network (Figure 1D). Low amplitude signals correspond to “normal activity” whilst high amplitude signals represent “epileptiform dynamics” (20). We use the quantity Brain Network Ictogenicity (*BNI*) (11, 15) to quantify the propensity of the network to generate seizures. In practice, *BNI* measures the average time that each node spends in epileptiform dynamics (*t*_*i*_, *i* = 1, …, *N*) over a sufficiently long computational time (we use 4 × 10^6^ time steps),

(3) $$\begin{array}{l} BNI=\frac{1}{N}\;\sum_{i=1}^{N}\frac{t_{i}}{T}. \end{array}$$

*BNI* varies between zero (all nodes display “normal activity” all the time) and one (all nodes show “seizure activity” all the time). The change in *BNI* upon removal of individual nodes or a set of nodes allows us to quantify their ictogenicity. Here, removal of nodes is implemented by setting all incoming and outgoing connections from and to other nodes to zero (Figures 1F–K). To measure the impact of removing a set of nodes on *BNI*, we define Set Ictogenicity (*SI*) as

(4) $$\begin{array}{l} SI_{X}=\frac{BNI^{\left(0\right)}-BNI^{\left(X\right)}}{BNI^{\left(0\right)}} \end{array}$$

where *BNI*^(0)^ is a reference value for the unperturbed network, and *BNI*^(*X*)^ denotes the *BNI* value after the removal of all *n* nodes in the subset *X* = {*x*_1_, .., *x*_*n*_}. We tune the parameter *K* in the model such that *BNI*^(0)^ equals 0.5 (11, 20) which means that on average the network spends half of the computational time in epileptiform dynamics. We consider this value as a useful reference because it enables us to study the result of network perturbations more efficiently (a realistic value of *BNI*^(0)^ would be much smaller, and changes of *BNI* upon node removals would be more difficult to measure). Note that for *n* = 1 in Equation (4), *SI*_*X*_ is equivalent to the Node Ictogenicity (*NI*_*X*_), previously introduced in Goodfellow et al. (11), which measures the effect of removing a single node in *BNI* (Figures 1F–H). *SI*_*X*_ is a succinct term for Δ*BNI*, which was introduced in Goodfellow et al. (11). Larger *SI*_*X*_ values denote greater contribution of the considered set *X* to seizure generation. *SI*_*X*_ takes a value of one when the removed set of nodes leads to elimination of epileptiform dynamics, whilst zero or negative values denote that removing those nodes did not reduce the network ictogenicity. In this study, we set all negative *SI*_*X*_ values to zero. More details about the calculation of *BNI* and *SI* are given in Supplementary Text S2.

### Exemplar Networks and Topological Properties

In order to explore and understand the effect of perturbations in different network structures, we first study *SI* in exemplar networks. We consider both directed and undirected artificial networks with random and “scale-free” [i.e., generated by the Barabasi-Albert algorithm (28) and the static model (29)] topologies, comprising 20 and 40 nodes. Exemplar networks are provided in Supplementary Figure 2. The choice of these networks is motivated by the existence of many different methods to derive networks from clinical data, which could give rise to different types of networks, including weighted, unweighted, directed and undirected networks. We choose random networks to serve as a baseline and scale free networks to better represent real-world networks. For example, iEEG networks have previously been shown to contain rich clubs, or hubs (which are a characteristic of scale free networks), and an analysis of the patient data used herein shows around half of networks to have an approximately scale free distribution in the network weights (see Supplementary Figure 3). We note that in contrast to the weighted functional networks inferred from patient data (see section Patient Information and Data), we used binary artificial networks for simplicity. We further consider common graph theory measures, such as the degree, betweenness centrality, clustering coefficient and eigenvector centrality (30, 31) to study how *SI* relates to these topological properties (eigenvector centrality was only computed for undirected networks, because it is undefined for directed networks). Further details for the graph theory measures are provided in Supplementary Text S3.

### Resection Strategies

In a network of *N* nodes there are $\left(\begin{array}{c} N\\ n \end{array}\right)$ distinct subsets of size *n*. Therefore, in order to evaluate how *SI* is distributed within a network of size *N*, and to definitively identify the set with the highest *SI*, one could use an exhaustive (or brute-force) search, which would require $\overset{N}{\underset{n=1}{\sum }}\left(\begin{array}{c} N\\ n \end{array}\right)$ calculations. We use such an approach herein to calculate the “ground truth” distribution of *SI*. For networks of size relevant for the study of the brain, the brute-force approach quickly becomes intractable. For example, a 40-node network exhaustive search would require 2^40^ − 1 ≈ 10^12^ calculations. We therefore need to develop computationally tractable methods for studying *SI*. Previous studies (11, 20) have used heuristic methods based on recursively adding a single node to build up an optimal set. One method, which we refer to as “simple ordering” (11) calculates *NI*_*X*_ for all possible single node removals (i.e., *N* initial calculations). Nodes are ranked according to their *NI*_*X*_ values and added sequentially to the set. Each time a new node is added, the *SI* of the new set is calculated, with termination when *SI* is greater than 0.99. An extension to this method, which we refer to as “recurrent ordering” recalculates the distribution of *NI* after the removal of each node to ensure that in every iteration the node with the highest *NI* of the perturbed network is added.

In addition to these heuristics, we test a resection strategy based on optimizing *SI* with a genetic algorithm. Genetic algorithms are stochastic search methods based on mimicking natural selection, in which an evolving population of candidate problem solutions is used to find an optimal solution (17, 32, 33). A typical genetic algorithm (GA) starts with a population that comprises candidate solutions (called individuals). Each individual is evaluated by a fitness (objective) function which quantifies how successfully the individual solves the problem. Based on the fitness scores, the genetic algorithm creates a new population of individuals by performing a number of stochastic genetic operations (i.e. crossover, mutation, selection), and keeps the best solutions generated (those that minimize the objective function). This process continues for multiple iterations (called generations) until convergence to an optimum solution is achieved (see Supplementary Figure 4). Multi-objective genetic algorithms (MOGAs) optimize the given problem for more than one objective function, returning Pareto optimal solution sets which represent the optimal trade-off between the objectives (32, 33). Here, we use the Non-dominated Sorting Genetic Algorithm-II (NSGA-II) (32, 34). Particularly, we use the implementation of the algorithm included in the MATLAB Global Optimisation Toolbox (version R2017b) and follow the optimization protocol from Avramidis et al. (32). We note that the optimization algorithm and code was adapted from Avramidis and Akman (35). Given that our purpose is to find the smallest set with the largest *SI*, we use two objective functions that minimize the number of nodes removed as well as the quantity 1−*SI*. After multiple generations, the algorithm returns optimal sets of nodes with different resection sizes. Due to the stochastic nature of the genetic algorithm, we execute eight independent runs initialized from a uniformly distributed set of random points in each case, and then utilize the convergence metrics from Avramidis et al. [(32) and references therein] to assess whether the algorithm is robust and reliable at identifying optimal sets with respect to different population sizes and generation numbers (i.e., the MOGA hyperparameters). In short, the convergence metrics evaluate the spread of the optimal sets in the two-dimensional plane of the objective functions. For efficiency, we set the genetic algorithm to discard sets of nodes larger than half of the network size by attributing them arbitrarily large objective values. The final MOGA parameter setting implemented in this work yielded consistent solutions across multiple runs, as quantified by the convergence metrics.

All the aforementioned search methods were evaluated on 20-node networks. In 40-node networks we did not use the “ground truth” strategy, given that it is computationally intractable to calculate *SI* for 2^40^ − 1 sets. We therefore introduce in this case an additional approach: a “random search” heuristic which picks at random a sample of sets of nodes for every resection size and takes as a solution the set with the maximum *SI*. For comparison purposes, we consider a sample size equal to the number of sets evaluated by the genetic algorithm across all generations. The number of samples per resection size was considered proportional to the logarithm of all possible sets of the respective size. This enabled a denser sampling at small resection sizes where the optimum solution is expected to be found.

### Comparison Between Resection Strategies That Are Based on Node and Set Ictogenicity

The ground truth is the only strategy that guarantees the detection of the most ictogenic set, because it is the only one that has access to the *SI* of all sets of nodes (Figures 1H,K,L). Thus, for the 20-node networks we compare all strategies to the optimum solution found from the ground truth. In order to evaluate how close a given solution is to the highest *SI* observed in the ground truth, we compute the Δ*SI*,

$$\begin{array}{l} \Delta SI=SI^{GT}-SI^{S} \end{array}$$

where *SI*^*GT*^ is the highest *SI* observed in the ground truth and *SI*^*S*^ is the *SI* of the optimum solution detected by a strategy *S* (for a given resection size). Our aim is to find a strategy that may find *SI*^*GT*^ (Δ*SI* = 0) while avoiding the inefficient and exhaustive search performed in the ground truth.

In the case of 40-node networks, we cannot calculate the ground truth and therefore we use the solution from the genetic algorithm (*SI*^*GA*^) as a reference to compare with other strategies,

$$\begin{array}{l} \Delta SI=SI^{GA}-SI^{s}. \end{array}$$

Note that in this case the Δ*SI* could be negative, because the genetic algorithm might be outperformed by another strategy.

### Patient Information and Data

We analyse intracranial electroencephalographic recordings from 20 patients (15 female, 5 male; median age 31 years, IQR 16 years, range 10–66 years) who underwent pre-surgical monitoring at Inselspital Bern. Thirteen of them were free from disabling seizures and auras for at least 1 year after surgery (Engel I), whereas the remaining seven did not show worthwhile improvement (Engel IV). Details regarding all patients included in this study are listed in Table 1. Before and after surgery, high resolution MRI images were acquired, as well as post-implantation CT images in order to identify the position of the implanted electrodes and the exact location of the resected brain tissue. Further details about this procedure can be found in Rummel et al. (10). An experienced epileptologist/electroencephalographer (K.S.) visually inspected all the recordings and identified the onset and termination of a representative seizure as well as any channels that had to be removed from the analysis due to the presence of permanent artifacts (<5% of channels). All signals were down-sampled to a sampling rate of 512 Hz, re-referenced against the median of all the artifact-free channels and band-pass filtered (forward and backward filtering to minimize phase distortion) between 0.5 and 150 Hz using a fourth-order Butterworth filter. All the patients gave written informed consent that their imaging and EEG data might be used for research purposes, and retrospective data analysis has been approved by the ethics committee of the Canton of Bern/Switzerland. In order to calculate functional networks using iEEG data we use a surrogate corrected version of mutual information (10, 24) which yields undirected networks (see Supplementary Text S1 and Supplementary Figure 1).

**Table 1:** Patient information.

**Patient**	**Engel class**	**Age at surgery**	**Gender**	**Syndrome**	**Etiology/MRI/Histology**	**Number of iEEG channels**	**Number of resected iEEG channels**
1	I	27	F	MTLE(R)	Non-lesional	64	20
2	I	58	F	MTLE(L)	Hippocampal sclerosis	64	13
3	I	27	M	LTLE(L)	Cluster of dysplastic neurons	56	5
4	I	36	M	PLE(L)	Low-grade glioma	74	13
5	I	19	F	MTLE(L)	Hippocampal sclerosis	42	11
6	I	25	F	FLE(R)	Non-lesional	98	11
7	I	27	M	TLE(L)	Non-lesional	60	11
8	I	27	F	PLE(R)	Non-lesional	68	13
9	I	10	F	MTLE(R)	Hippocampal sclerosis	37	9
10	I	20	M	MTLE(L)	Hippocampal atrophy	31	7
11	I	52	F	MTLE(R)	Hippocampal sclerosis	38	8
12	I	22	F	FLE(L)	Non-lesional	76	7
13	I	42	F	FTE(R)	Aneurysmal subarachnoid hemorrhage	80	6
14	IV	38	F	LTLE(L)	Dysplasia	59	2
15	IV	21	F	LTLE(L)	Meningitis	61	10
16	IV	59	F	MTLE(L)	Suspected amygdala dysplasia	49	8
17	IV	31	M	PLE(R)	Non-lesional	96	4
18	IV	31	F	FLE(R)	Tuberous sclerosis	36	3
19	IV	42	F	LTLE(L)	Temporo-basal dysplasia	24	6
20	IV	33	M	FLE(L)	Non-lesional	69	4

*Every row corresponds to a different patient*.

*MTLE, medial temporal lobe epilepsy; LTLE, lateral temporal lobe epilepsy; PLE, parietal lobe epilepsy; FLE, frontal lobe epilepsy; TLE, temporal lobe epilepsy; FTE, fronto-temporal epilepsy; R, right; L, left*.

## Results

The results section is arranged as follows. In order to better understand how sets of nodes within networks contribute to ictogenicity, we first study how *SI* is distributed across subnetworks of different sizes in artificially generated scale-free and random networks. We subsequently assess the relationship between *SI* and common graph theory metrics and test to what extent these metrics can predict the optimal set. We then apply a genetic algorithm and heuristics to the problem of finding the optimal set before finally testing the genetic algorithm on patient data.

### Set Ictogenicity in Different Network Topologies

In a network of size *N*, for a given subset (collection) of *n* nodes, *X* = {*x*_1_, .., *x*_*n*_}, where *n* < *N*, we use *SI*_*X*_ to quantify the reduction in ictogenicity that is achieved by removing all nodes in *X* from the network. To gain insight into how seizures arise in networks, we first seek to uncover what the relationship is between the obtained reduction in ictogenicity (*SI*_*X*_) and the number of nodes that are removed (*n*), and how this depends on network topology. For computational tractability we initially study 20-node artificially generated networks, considering the removal of up to ten nodes (i.e. up to half of the network). This network size is tractable for analysis using a brute-force approach and is relevant in the clinical context, where iEEG implantation schemes for some people may comprise around 20 electrodes, and investigations of standard clinical scalp EEG typically yields 19 channels.

**Figure 2:**
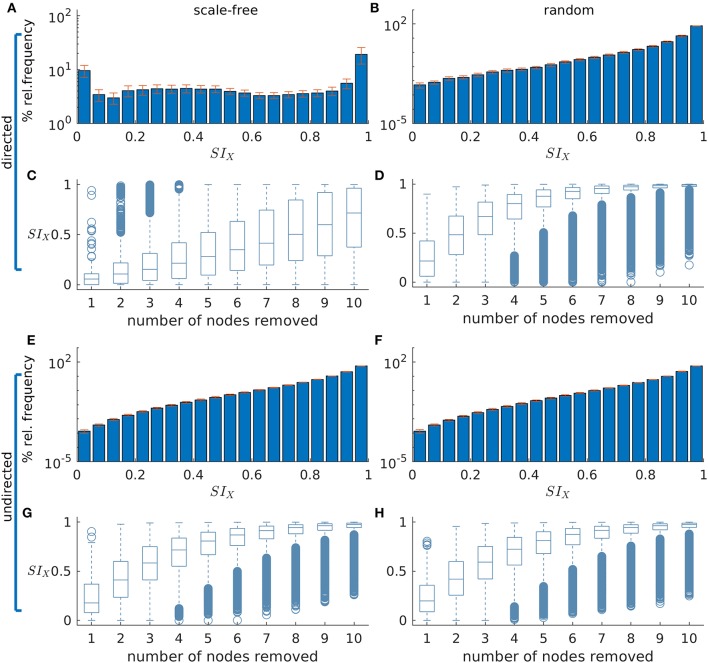
The distribution of Set Ictogenicity (*SI*) depends on network topology and resection size. **(A,B)** display the average *SI* distribution for ten directed artificial scale-free **(A)** and random **(B)** networks, respectively. Error bars denote the standard error across the ten network realizations. **(C,D)** show *SI* distributions split out by resection size (i.e., number of nodes removed) for the networks of panels **(A)** and **(B)**, respectively. **(E–H)** are similar to **(A–D)**, but for undirected networks. Parameters: network size *N* = 20; in the directed networks the in and out degree is 2, whilst in the undirected the mean degree is 2; scale-free degree distribution exponent γ = 3. The horizontal line in each boxplot indicates the median, while the bottom and top edges of each box are the 25th and 75th percentiles, respectively. The whiskers denote the range and circles the outliers.

Figures 2A,B,E,F demonstrate how *SI* is distributed in exemplar directed and undirected networks with scale-free and random topologies. We observe that the variance in *SI* is larger in the directed scale-free networks (Figure 2A) compared to random (Figures 2B,F) and undirected scale-free (Figure 2E) networks. Figure 2A shows that in the directed scale-free networks we studied, *SI* can take values between zero and one, depending on the set that is removed. Approximately 10% of sets do not reduce ictogenicity when removed. In addition, removal of 20% of the sets resulted in complete elimination of epileptiform dynamics (i.e., *SI*_*X*_ = 0). However, the effect of removal of the remaining sets is distributed approximately uniformly across *SI* values. In contrast, Figures 2B,E,F demonstrate that the *SI* distribution of random and scale-free undirected networks is more concentrated at high values, with very few sets having no effect on ictogenicity.

In Figures 2C,D,G,H *SI* is broken down by resection size. In all networks studied, the average *SI* increases as the size of the resected set increases. However, in the scale-free directed networks (Figure 2C) we studied, the relationship between resection size and average *SI* is approximately linear, and in random (Figures 2D,H) and undirected scale-free networks (Figure 2H), large average *SI* values are reached more readily for small set (resection) sizes. Furthermore, whilst in the directed scale-free networks we studied there are few sets with very large *SI*, in random and undirected scale-free networks there are instead few low *SI* values. We observe that typically, small resections have on average greater impact (i.e., higher *SI*) in random networks, compared to equivalent resections in directed scale-free networks.

### Set Ictogenicity and Graph Theory Measures

**Figure 3:**
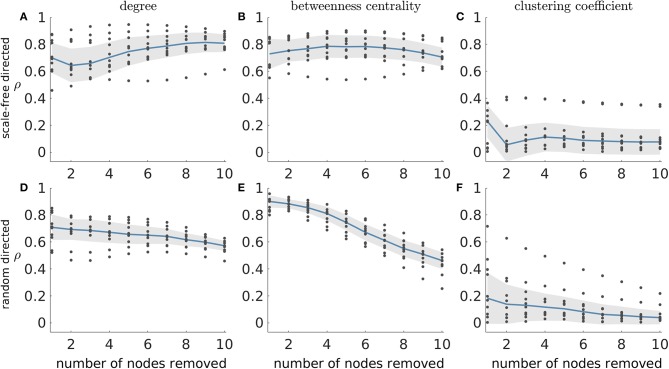
Absolute Spearman's correlation (ρ) between *SI* and average graph theory measures of the nodes in removed sets. **(A–C)** correspond to scale-free directed networks while **(D–F)** represent random directed networks. Each column shows ρ between *SI* and a different network measure: **(A,D)** average degree; **(B,E)** average betweenness centrality; and **(C,F)** average clustering coefficient of removed nodes. Ten network realizations were considered per network topology, hence the 10 dots for each resection size (i.e., number of nodes removed). The blue line represents the median across the network realizations and the shaded area displays the median absolute deviation. The parameters were the same as in Figure 2.

Figure 2 demonstrated that *SI* depends on network topology. To further understand this relationship, we investigated to what extent *SI* is related to common graph theoretic properties of nodes inside the sets selected for removal. We thus computed the Spearman's rank correlation between *SI* and the average degree, average betweenness centrality and average clustering coefficient of removed sets in directed scale-free and random networks. Figure 3 shows that *SI* is correlated with average degree and average betweenness centrality (median correlation larger than 0.6 for most resection sizes, see Figures 3A,B), but not with the average clustering coefficient in both scale-free and random networks (directed and undirected, see Supplementary Figure 7 for the latter). Analyzing different resection sizes we see that there are differences in correlation between directed scale-free networks and the other topologies we studied. In random networks, for example, the correlation between *SI* and average degree and betweenness centrality is high for small resection sizes but decreases for larger resections (Figures 3D,E). In contrast, correlation between degree and *SI* increases with resection size for directed scale-free networks (Figure 3A) and is relatively flat for betweenness centrality (Figure 3B). The low correlation for large resection sizes in random networks, particularly for betweenness centrality, is likely to be a consequence of the fact that most large sets have the same *SI* in random networks, as found in Figure 2D. Therefore, *SI* would be independent from measures of the constituent nodes.

### Identifying Optimal Resections

Having explored properties of *SI* distributions, we now turn to the problem of finding the set that optimally reduces ictogenicity upon removal, which is analogous to identifying the optimal resection in epilepsy surgery. Figure 3 demonstrated that the average degree and average betweenness centrality of removed sets are correlated with *SI*. This implies that these graph theory measures may potentially be used to find the sets with highest *SI*. We sought to test this by asking whether sets that produce maximum reductions in average degree or average betweenness centrality also produce maximal *SI* values. In order to identify maximal *SI* values, we calculated *SI* for all possible subsets of artificial networks with 20 nodes, which we refer to as the ground truth. Note that for a given network and resection size, there may exist multiple sets that produce maximal reductions in average degree or average betweenness centrality. We therefore calculated the average difference between the maximum *SI* (ground truth) and the *SI* of each set that yields maximum reduction of average degree and betweenness centrality (Δ*SI*). We henceforth denote sets that yield maximal reduction in average degree and average betweenness centrality as *SI*_deg_ and *SI*_*bet*_, respectively.

**Figure 4:**
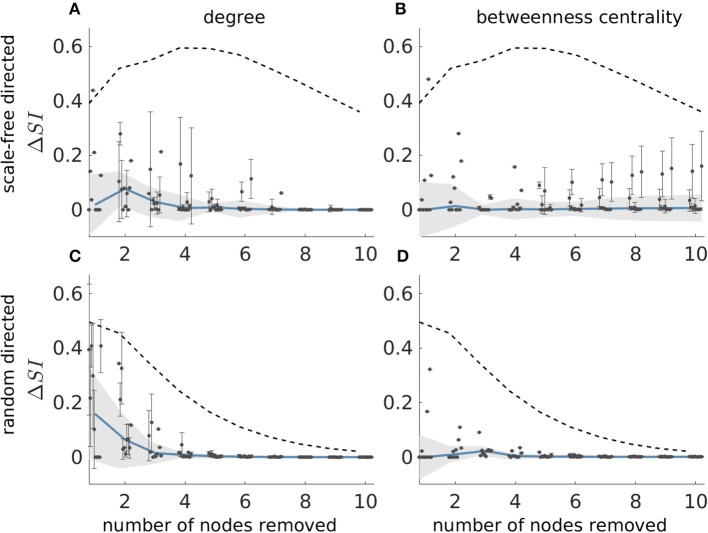
Difference between the *SI* value of the most ictogenic set as identified from the ground truth and the average *SI* of the sets which caused a maximal reduction in average degree **(A,C)**, and average betweenness centrality **(B,D)**, i.e., Δ*SI*, as a function of resection size. Error bars denote the standard deviation of the Δ*SI* values across the different sets that yield the maximal reduction in average degree or betweenness centrality when removed. The blue curve describes the median of the Δ*SI* values across 10 network realizations (black dots) and the shaded area their median absolute deviation (the dots are slightly shifted in the x-axis for better visualization). **(A,B)** Correspond to scale-free directed networks, whilst panels **(C,D)** to random directed networks. The dashed line denotes the average Δ*SI* between the *SI* of the ground truth most ictogenic set and the *SI* of all other possible sets (also averaged over the 10 network realizations). Parameters are the same as in Figure 2.

Figure 4 shows the results of this analysis for directed scale-free (Figures 4A,C) and random (Figures 4B,D) networks. We observe that in both scale-free and random directed networks, sets corresponding to *SI*_deg_ and *SI*_*bet*_ can yield *SI* values close to the optimal *SI* as defined by the ground truth (i.e., Δ*SI* = 0). In addition, the *SI*_deg_ and *SI*_*bet*_ sets always have lower Δ*SI* than the average *SI* from sets of the same size, which indicates that the reduction in average degree and average betweenness centrality are useful ways to find optimal sets (see the dashed line in the figure which represents the average Δ*SI* of every possible set of nodes). We also find that Δ*SI* gets smaller with increasing resection sizes, which is a consequence of how the distribution of *SI* changes with the resection size (i.e., as the number of nodes removed becomes large, *SI* becomes large in general, see Figure 2). However, we also observe that sets producing the same reduction in average degree or average betweenness centrality may have different *SI* values, as shown by the large error bars in Figures 4A,B. This means that information regarding the degree or centrality alone is insufficient to identify the set with the largest *SI*, since we would need further information to identify which of the sets corresponding to *SI*_deg_ and *SI*_*bet*_ would have largest *SI*. Furthermore, Δ*SI* may be quite large for some realizations of directed scale-free networks (see the existence of outliers in Figures 4A,B). In contrast, in random directed networks average degree and average betweenness centrality more accurately identify sets with optimal *SI* (see Figures 4C,D), particularly at larger resections (which is to be expected given that most sets yield *SI* close to the highest at large resection sizes independent of their constituent nodes). Results for undirected networks are shown in Supplementary Figure 8 where we also explored whether sets that produce maximum reduction in eigenvector centrality also produce maximal *SI* values. We also performed this analysis for clustering coefficient and found that the corresponding Δ*SI* values were very large which means that clustering coefficient was not able to identify the optimal set as defined by the ground truth.

### Alternative Strategies for the Identification of the Most Ictogenic Sets

Figure 4 showed that graph theory measures may often be used to identify the most ictogenic set. However, they may not be reliable for certain network realizations, particularly in directed scale-free networks. Therefore, to find the set of nodes with maximal ictogenicity we should calculate *SI*. However, calculating the *SI* for all possible sets is challenging computationally due to the large number of possible sets, particularly in large networks. Hence, in order to study larger networks, and to find optimal resections in general for practical applications, efficient methods are required to find sets with optimal *SI*. Here, we study two previously used heuristics, along with the NSGA-II genetic algorithm (32, 34). The heuristics we use are the simple ordering (11) and recurrent ordering (20) methods, which are based on the contribution of individual nodes to seizure generation. In contrast to building pseudo-optimal sets recursively, the genetic algorithm makes stochastic searches in the space of all possible sets of nodes using natural selection criteria.

**Figure 5:**
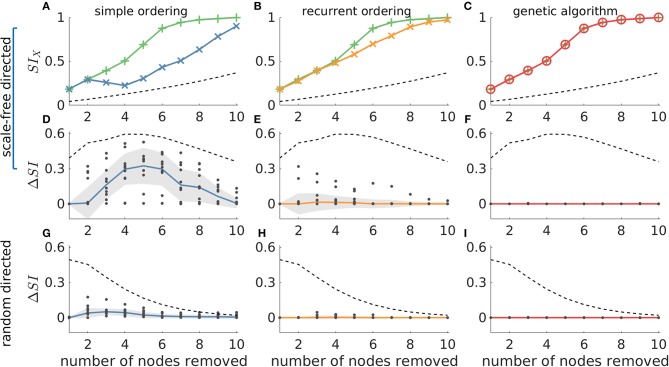
The genetic algorithm is the only resection strategy that identifies the most ictogenic set in all considered networks. The ground truth is compared to simple ordering (first column), recurrent ordering (second column) and the genetic algorithm (third column). **(A–C)** display *SI* values over the number of resected nodes in an exemplar artificial 20-node scale-free directed network, where the green lines represent the ground truth (i.e., the most ictogenic sets), whilst the blue, orange, and red lines display the *SI* of the sets found by each of the search strategies. The second and third rows show the Δ*SI* between the *SI* value of the ground truth and the set identified by each search strategy in scale-free and random directed networks, respectively. The dashed line represents the average *SI* (or Δ*SI*) of all possible combinations of nodes for each resection size and serve as a reference for comparison. The dots in **(D–I)** correspond to Δ*SI* values obtained for 10 different network realizations, the solid lines depict the median of Δ*SI* values, and the shaded area represents the median absolute deviation. Parameters were the same as in Figure 2. Additionally, the genetic algorithm was run for 100 generations with a population size of 200.

In Figure 5 we compare these methods against the ground truth in 20-node directed networks. We find that whilst both simple and recurrent ordering are able to identify solutions close to the highest *SI*, the genetic algorithm is the only approach that uncovers the optimal solution in all cases [the red line overlaps with the green line in Figure 5C, and Δ*SI* = 0 in panels (F) and (I)]. We further observe that in general the recurrent ordering provides better solutions than the simple ordering. These strategies performed differently for different network topologies, with scale-free directed networks being less amenable to the heuristic approaches than random networks [see the higher Δ*SI* values in Figure 5 panels (D) and (E) compared to (G) and (H)]. In undirected networks we observe similar results, though in this case both the genetic algorithm and the recurrent ordering strategy are able to find the sets with highest *SI* (see Supplementary Figure 9). We note that the genetic algorithm and the recurrent ordering approaches perform better than the heuristics based on graph theory measures (compare, for example, Figures 5E,H with Figure 4), further motivating the benefit of calculating *SI*. For all considered network topologies of this study, we ensured that the genetic algorithm converged across multiple independent realizations. The *SI* values of the optimal sets across multiple runs as well as the convergence metrics for exemplar networks can be found in Supplementary Figures 5, 6. The analysis above showed that the genetic algorithm can identify sets with the largest *SI* in all of the 20-node networks considered. In addition, the recurrent ordering strategy also found solutions relatively close to the optimum *SI* (see Figures 5E,H). Therefore, we sought to explore whether these optimisation strategies may also achieve good performances on larger, 40-node networks. For these larger networks, we restricted our analysis to directed scale-free networks given that they proved to be the more difficult to approach in our analysis of 20-node networks. Scale-free networks are characterized by the presence of hubs, which have been shown to exist in analyses of functional brain networks (20, 31, 36) and therefore they are more relevant to the clinical setting. Indeed, an analysis of the patient networks studied herein demonstrates that many of them have an approximately scale free distribution of weights (Supplementary Figure 3). As explained in Methods, we do not compute the ground truth in 40-node networks, since the number of all possible sets of nodes is too large. Instead, we use the genetic algorithm as a proxy for the ground truth and compare this to the simple and recurrent ordering heuristics. In addition, we employ a random search heuristic that searches through a solution space whose size is equal to the one of the genetic algorithm in order to test the uplift in performance of the latter compared to a stratified random approach.

**Figure 6:**
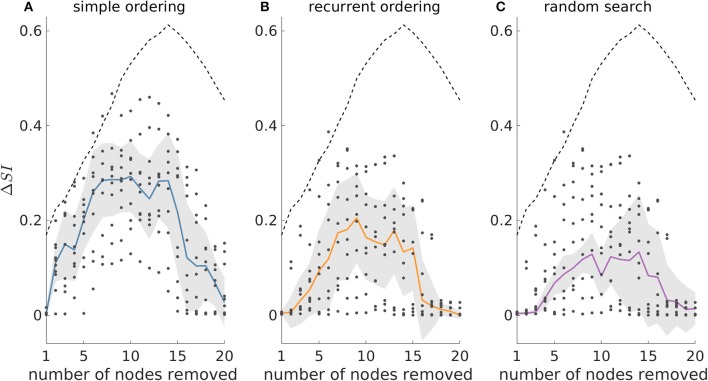
The genetic algorithm outperforms both simple and recurrent ordering as well as the random search heuristic in 40-node scale-free directed networks. Δ*SI* denotes the difference between the *SI* value of the optimal set as detected by the genetic algorithm and the *SI* solution found by **(A)** simple ordering, **(B)** recurrent ordering, and **(C)** random search across different resection sizes. The solid lines depict the median of the dots which correspond to the Δ*SI* values across 10 network realizations, whilst the shaded area illustrates the median absolute deviation. The dashed line represents the difference between the optimal set as detected by the genetic algorithm and the average of 20,000 random sets for each resection size (for resections up to three nodes, we considered all possible sets, since there were fewer than 20,000). Parameters: mean in and out degree equal to 4; population size 200, number of generations equal to 150.

Figure 6 shows that in the 40-node scale-free directed networks we studied, the genetic algorithm clearly outperforms all the other strategies. Note that in this figure Δ*SI* > 0 means that the genetic algorithm finds solutions with larger *SI* than the other approaches. We find that the uplift in performance of the genetic algorithm has a maximum at around sets of size 10 and then decreases for larger sets. This is due to larger resections being more likely to have *SI* = 1, as observed in our study of 20-node networks. This is further supported by the V-shaped dashed guideline curves corresponding to random removals which decrease for resection sizes larger than 15. In contrast, in undirected networks all approaches detect similar *SI* solutions (see Supplementary Figure 10).

### Identifying Optimal Resections in Patient Data

Our analysis of artificial networks demonstrates that the genetic algorithm is a good strategy for identifying sets of nodes with the largest *SI*. We therefore sought to test the application of the genetic algorithm to networks inferred from a cohort of 20 people with pharmacoresistant epilepsy who underwent resective surgery (see Materials and Methods). Using iEEG recordings, we constructed a functional network for each patient (see Supplementary Text S1 and Supplementary Figure 1) and used the genetic algorithm to identify the most ictogenic sets. Here, the network nodes correspond to iEEG channels, which in turn represent the brain tissue in the vicinity of the electrodes. Following (11) we defined the optimal set as the smallest set for which the *SI* exceeds 0.99. Note that as described in the Methods section, we executed multiple independent runs of the genetic algorithm, which is inherently stochastic, in order to obtain robust results. We observed that across the independent realizations we could obtain multiple optimal sets for a given patient (i.e., different sets of nodes with the same size and *SI* value).

**Figure 7:**
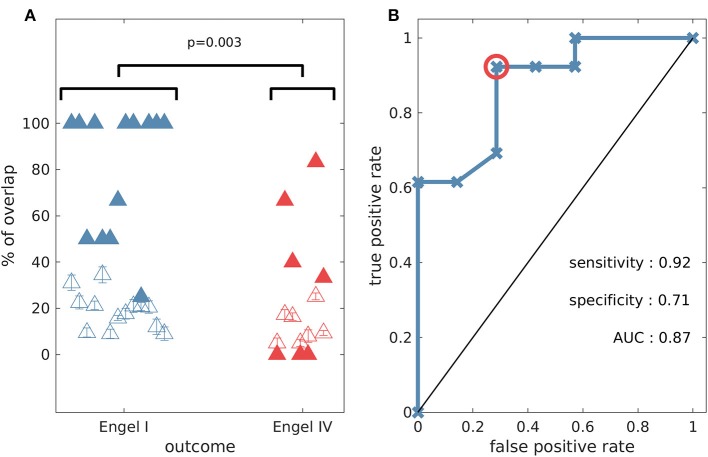
The percentage overlap between actual resections and model predictions is higher for patients with good outcome compared to those who had poor post-surgical outcome (one-sided Wilcoxon rank sum test *p* = 0.003). **(A)** Percentage overlap vs. patient surgical outcome grouped by Engel class (filled triangles). The unfilled triangles correspond to an average overlap between actual resections and equal-size random resections (100 random samples, and the error bars denote the standard error). **(B)** Receiver operating characteristic (ROC) analysis for Engel I (seizure free patients) vs. Engel IV (non-seizure free patients) using the percentage overlap as the classifier. The red circle indicates the point that is used for the calculation of sensitivity and specificity. Parameter setting for the genetic algorithm: population size equal to 200 and number of generations equal to 150.

In order to test the validity of predictions of the model, we compared them to the actual resections the patients underwent, and whether they were rendered seizure free as a result. Since the algorithm yielded multiple potential resections we calculated the overlap between the actual resected tissue and each of the optimal sets. Figure 7 shows the largest overlap per patient (for the minimal overlap see Supplementary Figure 11), with individuals grouped by post-surgical outcome. The overlap indicates the portion of nodes (electrodes) in the model suggestion that belongs to the actual resected tissue. We found significantly larger overlaps (one-sided Wilcoxon rank sum test, *p* = 0.003) for individuals who had good post-surgical outcome (Engel I) compared to those who had poor outcome (Engel IV) (see Figure 7A). In addition, we found that three out of the seven patients with poor post-surgery outcome presented zero overlap, meaning that our methods suggested completely different resections compared to those that were performed. We also calculated the overlap between the actual resected tissue and equal size random sets of nodes. We found that this overlap (Figure 7A—unfilled triangles) was smaller than the overlap between the actual resected tissue and our model suggestions (Figure 7A—filled triangles). The only exceptions were again the three cases having zero overlap with our predictions. Using the overlap as a classifier we found a sensitivity of 0.92, a specificity of 0.71, and an area under the curve (AUC) of 0.87 (see Figure 7B), which suggests our methods are reliable for classifying into outcome classes at the individual level. Interestingly, using graph theory measures alone Engel class I and IV patients could not be separated (Supplementary Figure 12). However, we found that if *SI* and the genetic algorithm are used to calculate the optimal size of the resection as a first step, eigenvector centrality and strength were able to separate the two groups well, whereas betweenness centrality could not (Supplementary Figure 13).

**Figure 8:**
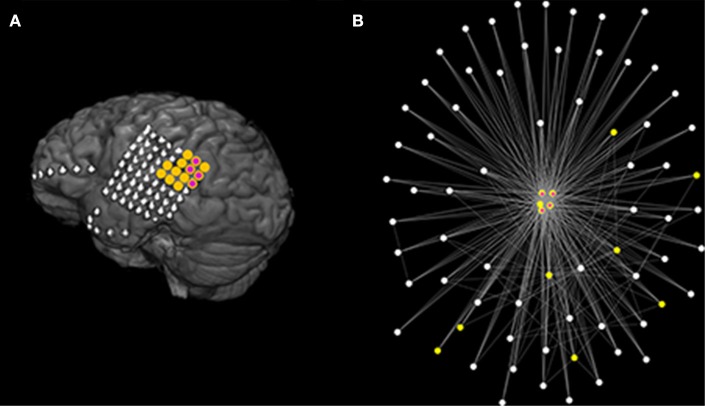
Actual clinical resection together with model suggestion using data from a good outcome patient. **(A)** Illustrates the MRI of a patient (fourth of Table 1) with good post-surgical outcome. The yellow color denotes the actual resected tissue while the purple color denotes the nodes suggested by the Genetic Algorithm. **(B)** Demonstrates the functional network as determined using the surrogate corrected mutual information. The network visualization maps strength of connectivity to distance in the plane. Colors are the same as **(A)**. Parameters are the same as those in Figure 7.

**Figure 9:**
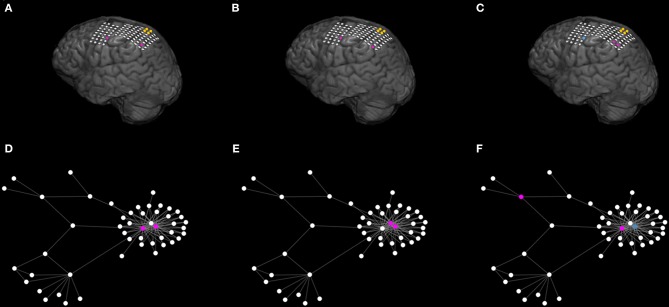
The genetic algorithm suggests multiple optimal sets and may further provide alternative sets under given restrictions. **(A–C)** Illustrate the MRI of a patient (seventeenth of Table 1) with poor post-surgical outcome. The yellow color denotes the actual resected tissue. **(A,B)** Exemplify two different (unconstrained) optimal sets (in purple) suggested by the Genetic Algorithm. **(C)** Shows an additional alternative optimal set (in purple) when constraining the genetic algorithm to avoid selecting the blue node. **(D–F)** Illustrate the corresponding functional network as inferred from the surrogate corrected mutual information. For illustrative reasons we only display the largest connected component of the network and connections characterized by interrelations larger than 0.09. The network visualization maps strength of connectivity to distance in the plane. Color coding is the same as **(A–C)**. The actual clinical resection [ yellow nodes of **(A–C)**] was not part of the displayed network component **(D–F)**. Parameters are the same as those in Figure 7.

Figure 8 illustrates the MRI and functional network of an Engel I patient (fourth of Table 1) where the optimal set suggested by the genetic algorithm belongs to the actual resected tissue. Figure 9 demonstrates the MRI and functional network of an Engel IV patient (seventeenth of Table 1). In this case, the genetic algorithm revealed two alternative optimal sets (Figures 9A,B,D,E). Here we demonstrate a further advantage of the genetic algorithm: it facilitates the avoidance of removing a certain node or nodes. This can be achieved by setting the objective function to a high value if the node(s) that should be avoided appear in a solution during the execution of the algorithm. Here, we avoid the selection of a highly ictogenic node (Figures 9C,F) and consequently the genetic algorithm substitutes it with another one. This constrained strategy of the genetic algorithm may be clinically valuable given that there may exist network nodes that cannot be removed due to their overlap with eloquent cortex, blood vessels or other anatomically indispensable areas.

## Discussion

In this study we used computational modeling and evolutionary optimization to understand how sets of nodes within a network contribute to its seizure generating capability (i.e., its ictogenicity). To do this we used a quantity called Set Ictogenicity (*SI*), which is a model-based quantification of the effect that removing a set of nodes has on the capability of a network to transition between healthy and epileptiform dynamics. We demonstrated that the way in which *SI* varies for different sets of nodes depends upon network topology. Whilst in exemplar 20-node random directed networks most sets of nodes have similar and large *SI*, in exemplar directed networks generated using the Barabasi-Albert model (i.e., “scale-free” networks), we observed a V-shaped distribution of *SI*. In the latter case, most sets do not yield a large reduction in ictogenicity when removed from the network. This difference is in part explained by the observed high correlation between *SI* and degree: since nodes in scale-free networks have higher degree variability, this leads to higher *SI* variability across sets of nodes. We further observed that *SI* is correlated with betweenness centrality. These results build upon our previous findings in reference (20), where we analyzed the correlation between graph theory measures and the Node Ictogenicity (which is the reduction in ictogenicity obtained by removing a single node). Our results add further evidence that targeting hubs, which would be sets of nodes with high average degree and betweenness centrality, is likely to be a good strategy for epilepsy surgery (20, 36–38). However, we have also shown that using graph theory measures alone is not guaranteed to provide the optimal resection. The use of models to link network topology to epileptiform dynamics and global search methods applied to the dynamics could therefore be important in clinical settings.

Several other studies have advocated the use of ideas from network theory and modeling to understand the ictogenic network and the effects of epilepsy surgery. Burns et al. (39), for example, studied the evolution of brain networks before, during and after seizures using iEEG data. They found that during seizure onset a group of electrodes relating to the seizure onset zone disconnects from the remaining network. Studies, such as these can provide insight into the nature of the ictogenic network and how it evolves during changes of brain state. The use of computational models to understand data such as these can help to provide a mechanistic understanding and potentially provide additional information not apparent in static analyses of networks. For example, we showed that although *SI* correlates with graph theory metrics, sets of nodes which induce maximum reduction in average degree or centrality are not necessarily sets with *the highest SI*.

Complementary to our approach, Jirsa at al. (13) have used the simulation platform “The Virtual Brain” to construct person specific models of seizures with the aim to identify the epileptogenic zone prospectively. In their model, structural connectivity is combined with hypotheses regarding the location of the seizure onset zone and seizure propagation patterns are compared to patient data. In our approach, we work with networks derived from iEEG and use the output of a model to explore which resections would optimally reduce the occurrence of abnormal brain activity. This does not assume *a-priori* information regarding which brain region may be responsible for initiating seizures. That is, we do not assume we know where the seizure onset zone is, rather we let the network and the model tell us which nodes should be removed. Indeed, we have shown using models that there can be a complicated relationship between the seizure onset zone and the epileptogenic zone (40).

We developed a global optimization framework that can be deployed in general to find the optimal resection, given a network structure. Epilepsy surgery relies on the identification of brain regions that are responsible for the emergence of epileptiform activity, but wherever possible mitigating effects on normal brain function. We therefore studied two objectives: the *SI* and the size of the resection, using a multi-objective genetic algorithm. Genetic algorithms are optimization methods based upon the process of evolution and natural selection. They have been widely applied in neuroscience (32, 41–43) and it has been shown that they are a valuable tool to solve combinatorial type computational problems (16, 17). Here, we showed that in small networks our genetic algorithm was able to find minimal sets with highest ictogenicity. In larger networks, we compared the genetic algorithm with other heuristic approaches and demonstrated that the genetic algorithm was always at least as good or better than these heuristics at finding the sets with highest ictogenicity.

Brain networks may be studied at different spatial and temporal scales using different data modalities (44). In the context of epilepsy surgery, most studies have focused on large-scale brain networks inferred from iEEG (45, 46) and MRI (47, 48). Network topology has been shown to evolve during seizures (49, 50), with structures changing from random to more regular in seizures and increasing randomness after seizure termination (51). Furthermore, it has been shown that hubs may play a crucial role in the generation of seizures (20, 52, 53). That is why here we studied both random and scale-free networks to build understanding about the epileptic brain. We found that the *SI* distribution varies in networks with different topologies and is more heterogeneous in directed scale-free networks compared to directed random networks. The framework we introduced is flexible and can be applied to smaller spatial scales, e.g. neuronal networks or smaller sized regions of interest in whole brain models (54, 55). At the smaller scale, these methods could shed light on, for example, why hubs of granule cells in the dentate gyrus are responsible for the emergence of seizures after brain injury (56). Use of these methods combined with experimental testing of perturbations, for example using optogenetics (57), could open up new avenues for targeted treatment for seizures (58).

We demonstrated the potential applicability of the genetic algorithm by applying it to functional brain networks derived from iEEG recordings of 20 patients who had undergone epilepsy surgery. We found that the model-derived optimal set had larger overlap with actual resections in the case of patients who were ultimately seizure free. This is in line with previous studies that have used computational models and heuristic approaches, based on properties of individual nodes, to test potential alternative resections (11, 12, 20). Furthermore, our framework achieved a classification performance comparable to recent studies that used machine learning and quantitative EEG methods (59). Depending on the way in which ictogenic networks are constructed, it is possible that in epilepsy surgery multiple nodes from the brain network are removed. We therefore here aimed to identify the indispensable brain region for seizure generation by considering the ictogenicity of sets of nodes. A particular advantage of the genetic algorithm that we demonstrated is that it naturally suggests multiple optimal sets that suppress the epileptiform activity of the network, due to the random nature of the search. These are alternative sets that give rise to optimal *SI* (see Figures 9A,B,D,E). Furthermore, there are natural ways to implement constraints, for example on nodes that should not be removed because they are essential for healthy brain function (Figures 9C,F).

There are a number of caveats to the approaches we outlined and opportunities to enhance the methodologies in the future. We validated the approach by measuring the overlap of the optimal set with actual resections, taking into account post-operative seizure freedom. Although the results of Figure 7 give confidence that the collection of nodes identified in the analysis were in fact ictogenic, it does not provide validation that the entire set itself is the optimal resection. Furthermore, the GA can provide multiple sets that are predicted to be effective in reducing seizures and these will be indistinguishable in our current model framework. In order to test these aspects further, we propose to work with experimental systems, whereby alternative resections can be performed (60).

We note that a large overlap between the model suggestion and the actual resected tissue was found in 2 cases in which outcome was Engel IV. In addition, in one Engel class I patient the overlap was small (see Figure 7A). Our approach assumes the existence of an ictogenic network and it has been shown that even focal epilepsies may involve widespread brain networks (46, 61–63). However, the electrodes are implanted in a designated brain region and the functional network that is inferred from them might not reflect the ictogenic network. Therefore, the initial placement of iEEG electrodes may be key here, and recent work has aimed to use modeling to uncover cases for which an alternative implantation scheme may be required (20). Future work should also aim to aid clinicians with regards to electrode implantation and to integrate different data modalities so that predictions may be more robust. We further note that although *SI* can be used to compare different resections, their actual values can be difficult to interpret. Linking *BNI* and *SI* to the rate of occurrence of epileptiform discharges in humans and experimental models will be an important avenue for future work that can aid the refinement of model predictions. We also note that the genetic algorithm is computationally more expensive than the other heuristics (see Supplementary Text S4), however, it may be further optimized in the future by making use of parallelization and GPUs (32), for example. In addition, employing a global search method allows one to incorporate constraints that may be useful in the clinical setting. For example, one could introduce a penalty for large spatial spread of nodes in the optimal set, therefore identifying optimal contiguous regions of tissue for resections in practice.

In conclusion we presented a computational approach that quantifies the contribution of brain regions to seizure generation. Our approach enhances the understanding of how perturbations in brain networks may lead to seizure freedom. It allows multiple surgical strategies to be tested *in silico* in order to find the optimal set that reduces the network ictogenicity. In addition, the genetic algorithm that we deployed finds the optimal trade-off between the size of the resected tissue and the reduction in network ictogenicity. Our results show promise that the computational approaches introduced herein have the potential to be incorporated into surgery decision pipelines in practice.

## Data Availability Statement

The synthetic networks are available upon request.

## Ethics Statement

All the patients gave written informed consent that their imaging and EEG data might be used for research purposes, and retrospective data analysis has been approved by the ethics committee of the Canton of Bern/Switzerland.

## Author Contributions

PL, MG, MR, KS, and CR: conceptualization. EAb, CR, and KS: data curation. PL and CR: formal analysis. MG: funding acquisition. PL, ML, MG, MM, CR, EAv, and OA: methodology. MG: project administration. PL, EAv, ML, MM, and CR: software. MG: supervision. PL, ML, and MG: writing-original draft preparation. PL, EAv, ML, EAb, MM, OA, MR, CR, KS, and MG: writing-review and editing.

### Conflict of Interest

The authors declare that the research was conducted in the absence of any commercial or financial relationships that could be construed as a potential conflict of interest. The reviewer MM and handling Editor declared their shared affiliation at the time of review.
